# Dynamics in cognition and health-related quality of life in grade 2 and 3 gliomas after surgery

**DOI:** 10.1007/s00701-022-05408-2

**Published:** 2022-11-04

**Authors:** Teodor Svedung Wettervik, Åsa A. Munkhammar, Malin Jemstedt, Marcus Ersson, Francesco Latini, Mats Ryttlefors, Maria Zetterling

**Affiliations:** Section of Neurosurgery, Department of Medical Sciences, Uppsala University, SE-751 85 Uppsala, Sweden

**Keywords:** Astrocytoma, Brain tumor, Cognition, Health-related quality of life, Low-grade glioma, Oligodendroglioma

## Abstract

**Background:**

The focus of clinical management and research in gliomas has been on survival, but the interest in the treatment effects on cognition and health-related quality of life (HRQoL) is emerging. The primary aim of this study was to investigate the dynamics in cognition after brain tumor surgery for astrocytomas and oligodendrogliomas grade 2 and 3. The secondary aim was to investigate the association of postoperative changes in cognition with changes HRQoL.

**Methods:**

In this observational study, 48 patients operated for an astrocytoma or oligodendrogliomas, grade 2 or 3, at the Department of Neurosurgery, Uppsala, Sweden, 2016–2021, were included. Cognitive and language skills were assessed with a selected test battery and HRQoL was patient-reported as assessed with RAND-36 pre- and approximately 3 months postoperatively.

**Results:**

There was a significant postoperative decrease in attention span and verbal learning, but the patients improved in the test for visual memory. There was no change in visual attention, executive function, verbal memory, visual organization and construction, verbal fluency, and confrontation naming. The RAND-36 variables physical function, role physical, general health, vitality, and social functioning decreased significantly after surgery. Patients operated for tumor recurrence exhibited greater deterioration in attention and a greater extent of resection correlated with a less pronounced decrease in verbal memory, but there were otherwise weak associations between the dynamics in cognition and patient-, tumor-, and treatment-variables. A decline in cognitive variables was not associated with worse HRQoL.

**Conclusions:**

Although both several cognitive and HRQoL domains deteriorated postoperatively, these changes did not correlate with each other. This highlights the complexity of cognitive and HRQoL dynamics in the early postoperative phase.

**Supplementary Information:**

The online version contains supplementary material available at 10.1007/s00701-022-05408-2.

## Introduction

The development of better treatment strategies, including early, maximal, and safe surgery and adjuvant oncological therapies, has led to a significant improvement in survival with several years, for patients with diffuse gliomas grade 2 and 3 [9, 38]. However, these tumors typically affect seemingly asymptomatic young adults and are often located in eloquent cortical and subcortical brain areas involved in, e.g., language, sensorimotor, and visual functions [5]. Hence, along with the improvements in survival with better treatment strategies, concerns have been raised regarding preservation of a cognitive abilities and health-related quality of life (HRQoL). The concept of onco-functional balance has been introduced, which takes into account that the gain in survival with increased extent of resection (EOR) must be weighed against the concurrent risk of inflicting any neurological sequelae [18]. There are several techniques to make surgical treatment safer. Preoperative anatomical and functional imaging, intraoperative neurophysiology, and functional monitoring during awake surgery contribute with vital information regarding the eloquent and non-eloquent surgical areas [6, 34]. However, even with the best pre- and intraoperative settings available, functional preservation cannot always be guaranteed [46]. Even awake intraoperative monitoring has limitations due to technical, spatial, and time constraints. Particularly, it is difficult to fully assess complex, higher-order functions [8] and patients with lower grade diffuse gliomas typically exhibit decreased cognitive abilities and HRQoL during their course of disease, both due to the disease itself as well as treatments including surgery, radiation, and chemotherapy [21, 29, 41]. Research on the risk of functional aspects including cognition and HRQoL of diffuse gliomas is still limited, but of great importance to reveal potentially negative effects of surgery in patients who experience no focal neurological deficits, but still do not recover well.

The primary aim of this study was to determine the dynamics in cognition and language before and after surgical treatment of diffuse gliomas grade 2 and 3. The secondary aim was to study the association of postoperative changes in cognition with patient-, tumor-, and treatment-variables as well as with changes in patient-reported HRQoL.

## Materials and methods

### Patients

Adult patients (≥ 18 years of age) with a suspected diffuse glioma grade 2 and 3 operated at the Department of Neurosurgery, Uppsala University Hospital, during the period 22 August 2016 to 1 May 2021 were eligible for inclusion. Out of 123 patients with a suspected glioma, those 48 patients with an astrocytoma (IDH mutation without 1p19q co-deletion) or oligodendroglioma (IDH mutation in combination with 1p19q co-deletion), grade 2 or 3, with both pre- and postoperative HRQoL and neuropsychological cognitive assessment were included (Supplementary Fig. 1).

### Demographics, surgery, postoperative care, adjunct treatments, and follow-up

Demographic and treatment data were collected from the medical records.

As described in previous studies by our group [13, 39], the intent of the tumor surgery was maximal and safe resection. The surgical procedure included craniotomy and microsurgical technique, guided by neuronavigation and intraoperative ultrasound. For tumors located in or near motor cortex/pathways, intraoperative neurophysiological monitoring of motor function was performed. For tumors close to eloquent areas related to language function or other cognitive domains, awake surgery was done to monitor those functions, as previously described [13]. Further follow-up and eventual adjunct oncological treatments (radio- and/or chemotherapy) were discussed and decided on at multidisciplinary conferences after the histomolecular diagnosis had been determined, in accordance with the European Association of Neuro-Oncology (EANO) guidelines [43]. The patients were assessed by a neurosurgeon, speech therapist, and neuropsychologist approximately at 3–5 months postoperatively.

### Radiology

A magnetic resonance imaging (MRI) scan was done prior to surgery and within 48 h after the surgery in accordance with our glioma imaging protocol [7]. Location and radiological features of the tumors were assessed with volumetric T1W, T2W, and T2-FLAIR [7, 39]. T2 turbo spin echo or T2-FLAIR images in Vue picture archiving and communication system (PACS) software (version 11.1.0, Carestream Health Inc., Rochester, NY) were used to segment the lesions both pre- and postoperatively with the aid of a semiautomatic method (Livewire Algorithm) [14]. Tumor location and eloquence was analyzed by means of diffusion tensor imaging (DTI) reconstructions, as described in detail in a previous study by our group [13]. The tumors were considered eloquent if they exhibited infiltration of the white matter pathways in the inferior fronto-occipital fascicle (IFOF), arcuate fasciculus (AF), corticospinal tract (CST), and optic radiation (OR), i.e., the “minimal common brain” [35].

### Cognitive, language, and health-related quality of life assessment

Cognitive and language abilities were assessed by a licensed neuropsychologist and speech therapist pre- and approximately 3–5 months postoperatively, using a selected battery of tests, as described in detail in Supplementary Table 1. In brief, both Trail Making Test (TMT) trials 2 and 4 reflect the capacity for visual attention and scanning, but TMT 4 also includes mental flexibility and divided attention [3]. Digit span forward reflects attentional capacity and coding reflects attention as well as visuomotor processing speed [28, 42]. Auditory Verbal Learning Test (AVLT) reflects verbal learning and memory [30]. Rey Complex Figure Test (RCFT) copy trial and delayed recall reflect visual organization and construction as well as visual memory [25]. Boston Naming Test (BNT) [10] reflects naming ability and Controlled Oral Word Association (COWA) [1] reflects verbal fluency; however, these tests were mostly done in patients with left-sided tumors. The test results were reported as *z*-scores, taking into account age, sex, and the level of education in relation to a normal population. RAND-36 is a generic measure of HRQoL, which is available in Swedish [24]. RAND-36 is based on 36 questions that cover eight HRQoL domains including physical functioning (PF), role physical (RP), bodily pain (BP), general health (GH), vitality (VT), social functioning (SF), role emotional (RE), and mental health (MH). The scales range from 0 (worst possible) to 100 (best possible). The RAND-36 questionnaire was filled in within a few weeks to 1 day prior to surgery and approximately 3–5 months postoperatively.

### Statistical analysis

The difference in the cognitive test results and the RAND-36 variables pre- and postoperatively was calculated with the Wilcoxon signed-rank test. The association between dynamic changes in the cognitive tests with demographics, tumor characteristics, treatments, and dynamic changes in the HRQoL-variables were analyzed with the Spearman rank correlation test. BNT and COWA were not explored in these analyses since only a smaller subset of the patients had performed these tests (*n* = 22). A principal component analysis was also conducted, including the dynamic changes in the cognitive tests and HRQoL-variables. A *p* value < 0.05 was considered statistically significant. We abstained from adjustments for multiple comparisons, since this was an exploratory study. The statistical analyses were performed in SPSS version 27 (IBM Corp, Armonk, NY, USA).

## Results

### Demography, tumor characteristics, treatments, and acute complications

In the study cohort of 48 glioma patients, median age was 38 (IQR 31–45) years and the male/female ratio was 34/14 (71/29%), as shown in Table 1. The majority of patients were operated on for the first time (*n* = 36 (75%)) and 12 (25%) due to tumor recurrence. Preoperative tumor volume was in median 31 (IQR 10–57) mL, the tumor was most often located on the left side (*n* = 29 (60%)), and in an eloquent brain area in 41 (85%) of the cases. The tumor type was astrocytoma grade 2 in 15 (31%) and grade 3 in 12 (25%) cases and oligodendroglioma grade 2 in 14 (29%) and grade 3 in 7 (15%) cases. The median preoperative tumor volume was 31 (IQR 10–57) mL, 52% were operated on awake (48% under general anesthesia), and the median EOR was 91 (IQR 84–100) %. Thirty-one (65%) patients received oncological treatment before the postoperative cognitive tests; twelve (25%) radiation alone, 5 (10%) chemotherapy alone, and 14 (29%) radiochemotherapy.

**Table 1:** Demographics, tumor characteristics, and surgical variables

Patients, *n* (%)	48 (100%)
Age, median (IQR)	38 (31–45)
Sex (male/female), *n* (%)	34/14 (71/29%)
Education (elementary/high-school/university), *n* (%)	1/32/13 (2/67/27)
Tumor volume (ml), median (IQR)	31 (10–57)
Tumor eloquence (yes/no), *n* (%)	41/7 (85/15%)
Tumor lateralization (right/left), *n* (%)	19/29 (40/60%)
New tumor/tumor residual, *n* (%)	36/12 (75/25%)
Surgery awake/sedated, *n* (%)	25/23 (52/48%)
Extent of resection (%), median (IQR)	94 (81–100)
Tumor type	
Astrocytoma, grade 2, *n* (%)	15 (31%)
Astrocytoma, grade 3, *n* (%)	12 (25%)
Oligodendroglioma, grade 2, *n* (%)	14 (29%)
Oligodendroglioma, grade 3, *n* (%)	7 (15%)
Postoperative oncological treatment before cognitive and RAND-36 evaluation, *n* (%)	31 (65%)

*IQR* interquartile range

Eighteen (38%) patients exhibited at least one new-onset neurological symptom within the first 7 post-operative days. Eight 8 (17%) developed a paresis, 9 (19%) dysphasia, 7 (15%) seizure, and 4 (8%) the supplementary motor area (SMA) syndrome [26]. Only 4 (8%) patients had persistent sequelae based on clinical neurological exam by the neurosurgeon and 10 (21%) in total had exhibited seizures at some point postoperatively until the 3 month-follow-up. Thirty-three (69%) patients had returned to work for 25% or more at 1 year.

### Pre- and postoperative cognitive and health-related quality of life assessments

**Fig. 1:**
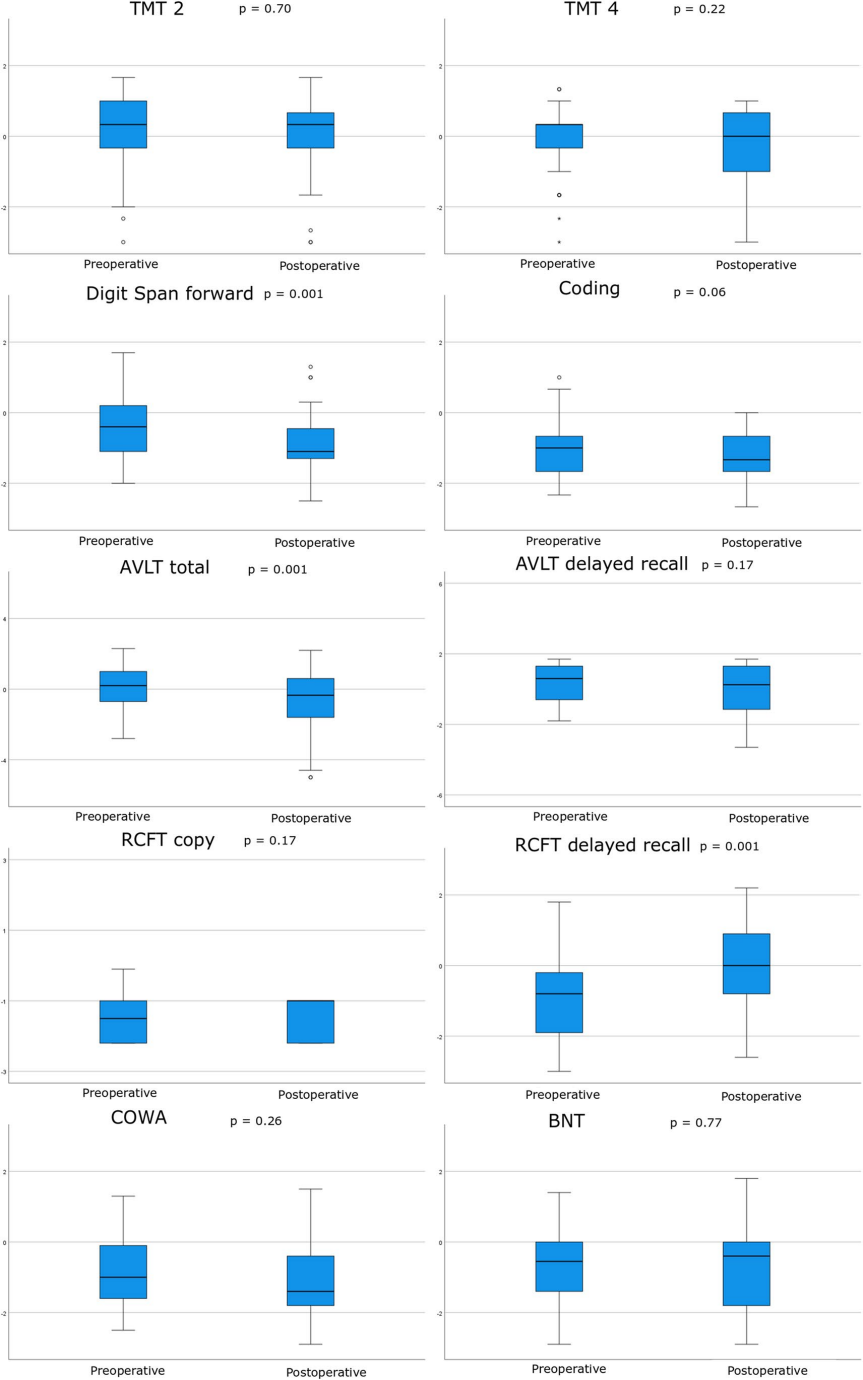
Cognitive domains and language before and after surgery for diffuse gliomas grade 2 and 3

The preoperative cognitive and HRQoL assessments were conducted the day before surgery or in the outpatient clinic 1 month before and postoperatively in median 3 (IQR 3–5) months after surgery. As demonstrated in Table 2 and Fig. 1, the patients performed worse cognitive test results postoperatively for digit span forward (*p* = 0.001) and AVLT total (*p* = 0.001), whereas they did better in RCFT delayed recall (*p* = 0.001). There was no significant change in TMT 2 and 4, coding, AVLT delayed recall, RCFT delayed recall, COWA, and BNT score after surgery. In total, deterioration with − 1 *z*-score or more occurred for 17% in TMT 2, 21% in TMT 4, 21% in digit span forward, 13% in coding, 31% in AVLT total, 22% in AVLT delayed recall, 3% in RCFT copy, 3% in RCFT delayed recall, 29% in COWA, and 9% in BNT. Correspondingly, improvement with + 1 *z*-score or more occurred for 19% in TMT 2, 10% in TMT 4, 3% in digit span forward, 9% in coding, 3% in AVLT total, 12% in AVLT delayed recall, 17% in RCFT copy, 43% in RCFT delayed recall, 14% in COWA, and 5% in BNT.

**Table 2:** Cognitive capacity before and after glioma surgery—a Wilcoxon signed-rank test

Cognitive variables	Preoperative	Postoperative	Paired difference ∆(postoperative-preoperative)	*p* value
TMT 2	0.33 (− 0.33–1.0)	0.33 (− 0.33–0.67)	0.00 (− 0.67–0.42)	0.70
TMT 4	0.33 (− 0.33–0.33)	0.00 (− 1.00 to − 0.67)	0.00 (− 0.67–0.33)	0.22
Digit span forward	− 0.40 (− 1.10–0.20)	− 1.10 (− 1.30 to − 0.40)	− 0.70 (− 0.80–0.00)	***0.001***
Coding	− 1.00 (− 1.67 to − 0.33)	− 1.33 (− 1.67 to − 0.67)	0.00 (− 0.67–0.00)	0.06
AVLT total trials I-V	0.10 (− 0.98–0.98)	− 0.35 (− 1.60–0.65)	− 0.60 (− 1.28 to − 0.13)	***0.001***
AVLT delayed recall	0.30 (− 0.60–1.30)	− 0.25 (− 1.18–1.30)	− 0.15 (− 0.70–0.30)	0.17
RCFT copy trial	− 1.70 (− 2.20 to − 1.10)	− 1.00 (− 2.20 to − 1.00)	0.00 (0.00–0.63)	0.17
RCFT delayed recall	− 0.85 (− 1.90 to − 0.20)	0.00 (− 0.83–0.90)	0.60 (0.10–1.80)	***0.001***
COWA (FAS version)	− 0.95 (− 1.55–0.25)	− 1.15 (− 1.83 to − 0.30)	− 0.20 (− 1.30–0.40)	0.26
BNT	− 0.40 (− 1.40–0.00)	− 0.40 (− 1.80–0.00)	0.00 (− 0.40–0.40)	0.77

The variables are described as *z*-scores and the table shows medians and interquartile ranges. Bold and italics indicate statistical significance

In HRQoL (Supplementary Table 2), the glioma patients deteriorated significantly in PF (*p* = 0.002), RP (*p* = 0.001), GH (*p* = 0.009), VT (*p* = 0.02), and SF (*p* = 0.001), but no change was found in BP, RE, and MH.

### Explanatory variables for a change in pre- and postoperative cognitive test results

The association between changes in cognitive test results in relation to demographic, tumor-, and treatment-related variables are demonstrated in Table 3. A greater deterioration in coding correlated with female sex (*r* =  − 0.34, *p* < 0.05), tumor recurrence rather than primary tumor surgery (*r* =  − 0.48, *p* < 0.001), awake surgery (*r* =  − 0.34, *p* < 0.05), and a lower chance to return to work 1 year after surgery (*r* = 0.33, *p* < 0.05). A greater deterioration in AVLT delayed recall correlated with a smaller extent of resection (*r* = 0.56, *p* < 0.001) and development of postoperative neurological symptoms (*r* =  − 0.38, *p* < 0.05). In addition, a worse decline in RCFT delayed recall correlated with receiving additional oncological treatment before the cognitive tests (*r* =  − 0.42, *p* < 0.05). Otherwise, there were no significant associations between the dynamic changes in the cognitive tests and the potential explanatory variables.

**Table 3:** Postoperative dynamics in cognitive abilities in relation to demographics, tumor characteristics, and surgical variables—a Spearman correlation analysis

Variables	∆TMT 2	∆TMT 4	∆Digit span forward	∆Code	∆AVLT total	∆AVLT delayed	∆RCFT copy	∆RCFT delayed
Age	− 0.20	− 0.27	− 0.21	− 0.02	− 0.03	− 0.33	− 0.24	− 0.01
Sex^1^	− 0.15	− 0.20	0.20	*** − 0.34***^***a***^	0.04	0.05	0.14	0.18
Education^2^	0.08	− 0.04	0.07	− 0.08	− 0.19	− 0.04	− 0.01	0.23
Eloquence^3^	− 0.25	− 0.15	− 0.10	− 0.20	− 0.07	− 0.29	− 0.02	− 0.02
Tumor lateralization^4^	− 0.25	− 0.10	− 0.10	− 0.14	− 0.23	− 0.12	0.11	− 0.20
Tumor recurrence^5^	− 0.23	− 0.04	− 0.14	*** − 0.48***^***b***^	0.11	− 0.10	0.22	− 0.28
Tumor volume	− 0.20	− 0.12	− 0.03	− 0.08	− 0.11	− 0.29	− 0.05	− 0.09
Awake surgery^6^	− 0.27	− 0.24	− 0.20	*** − 0.34***^***a***^	− 0.20	− 0.16	− 0.10	− 0.22
Extent of resection	− 0.01	− 0.02	− 0.01	0.17	0.16	***0.56***^***b***^	− 0.07	− 0.17
Postoperative complication^7^	− 0.17	− 0.22	− 0.03	− 0.09	− 0.35	*** − 0.38***^***a***^	− 0.09	0.14
Oncological treatment before follow-up^8^	− 0.05	− 0.17	0.14	− 0.12	0.15	0.00	− 0.33	*** − 0.42***^***a***^
Return to work at 1 year^9^	0.10	0.22	0.00	***0.33***^***a***^	0.23	0.21	− 0.02	0.08

^1^Male = 0, female = 1. ^2^Considered ordinal; elementary school = 0, high-school = 1, university = 2. ^3^No = 0, yes = 1. ^4^Right hemisphere = 0, left hemisphere = 1. ^5^No = 0, yes = 1. ^6^No = 0, yes = 1. ^7^No = 0, yes = 1. ^8^No = 0, yes = 1. ^9^No = 0, yes = 1

^a^*p* value < 0.05, ^b^*p* value < 0.001. Bold and italics indicate statistical significance

### Relation between dynamic changes in cognition and health-related quality of life

The association between changes in cognitive test results in relation to changes in HRQoL variables is demonstrated in Table 4. A greater deterioration in digit span forward (*r* =  − 0.44, *p* < 0.01) and RCFT copy (*r* =  − 0.37, *p* < 0.05), respectively, were associated with improvement/less pronounced deterioration in RP. There were otherwise no association between the changes in cognitive test results and the HRQoL-variables. In a PCA loading plot (Supplementary Fig. 2), the association between the cognitive and HRQoL-variables was low. However, the Kaiser–Meyer–Olkin test was only 0.26, indicating that the reliability of this analysis was also low.

**Table 4:** Postoperative dynamics in cognitive abilities in relation to health-related quality of life—a Spearman correlation analysis

Variables	∆TMT 2	∆TMT 4	∆Digit span forward	∆Code	∆AVLT total	∆AVLT delayed	∆RCFT copy	∆RCFT delayed
∆PF	− 0.02	0.14	0.01	0.07	0.02	− 0.14	− 0.19	0.21
∆RP	− 0.18	− 0.12	*** − 0.44***^***b***^	− 0.02	0.01	− 0.29	*** − 0.37***^***a***^	0.08
∆BP	− 0.14	− 0.04	− 0.15	− 0.03	− 0.21	− 0.33	− 0.31	− 0.14
∆GH	− 0.12	0.01	0.07	− 0.19	0.28	− 0.15	− 0.16	− 0.17
∆VT	− 0.17	0.02	− 0.13	− 0.25	− 0.04	− 0.21	0.12	0.08
∆SF	0.08	0.13	− 0.15	0.06	0.01	− 0.27	− 0.21	− 0.13
∆RE	0.01	0.20	− 0.31	− 0.04	− 0.25	− 0.20	0.19	− 0.34
∆MH	− 0.02	− 0.18	− 0.27	− 0.16	− 0.14	− 0.09	0.29	− 0.20

∆ = postoperative value − preoperative value of the specific variable

^a^*p* value < 0.05, ^b^*p* value < 0.01. Bold and italics indicate statistical significance

## Discussion

In this study, including 48 patients with diffuse gliomas grade 2 and 3 with cognitive and HRQoL data from the pre- and postoperative phase, we found that many cognitive and HRQoL domains deteriorated postoperatively. Although this was a small study, it was still surprising that the patient-, tumor-, and surgery-related variables exhibited such weak associations with postoperative changes in cognition. Similarly, although both several cognitive and HRQoL domains deteriorated postoperatively in the entire cohort, these changes correlated very weakly with each other. Our study highlights the complexity of cognitive and HRQoL dynamics in the early postoperative period and that more follow-up and active rehabilitation may be warranted during this phase.

### Dynamics in pre- and postoperative cognitive test results

We found a significant postoperative decline in the cognitive scores for attention span (digit span forward) and verbal learning (AVLT total). There was also a trend towards slower visuomotor processing speed (coding), whereas the test scores for visual attention and mental flexibility (TMT 2 and 4) and verbal expression (BNT and COWA) were stable. The test scores improved regarding visual memory (RCFT delayed recall). However, the RCFT has a low test–retest reliability and a significant practice effect is often seen on follow-up tests, which may persist for months [11]. Therefore, we are cautious to interpret this as an improvement in cognitive functioning rather than a mere practice effect.

There are only a limited number of previous studies on the cognitive dynamics after diffuse glioma surgery. Our findings corroborate those from several previous publications, indicating a general cognitive postoperative decline after lower grade glioma surgery [23, 31, 45], although others have reported no decline [2, 15] and even improvements [40]. However, a major challenge when comparing these studies is patient and treatment heterogeneity. First, some studies have also included glioma grade 4 patients [40]. Second, others have studied glioma grade 2 patients with specific tumor locations, e.g., only limited to the temporal lobe [23] or insula [45]. Third, some series included only awake surgical cases [15]. Fourth, the exact test batteries as well as the time point (from the first days to several months postoperatively) of cognitive assessment have varied substantially between the studies [2, 23, 40, 45]. Although our study also carries inherent flaws regarding patient and treatment heterogeneity, the available reports in the literature are still limited and our study contributes with further data on early dynamics of cognition within the first months following surgery for diffuse gliomas grade 2 and 3. These data may provide further insights on the early clinical course after surgery in diffuse glioma patients.

### Cognition before and after glioma surgery—explanatory variables

The statistical analyses of explanatory variables for dynamic changes in the cognitive tests should be assessed cautiously considering the relatively low number of patients and that they were unadjusted for multiple comparisons. It was still surprising that the patient-, tumor-, and treatment-related variables were so weakly associated with the dynamic changes in cognition. There were only a few stronger (*p* < 0.001) associations. There was a greater decline in processing speed for those operated due to tumor recurrence rather than primary surgery. This may reflect that patients with tumor recurrence exhibited a more exhausted capacity for neuroplasticity after some years with the disease and after previous surgical and oncological treatments. In addition, those operated with a greater EOR had a lower rate of decline in verbal learning (AVLT total). A greater EOR could reduce the negative impact of the tumor on these cognitive networks. The association could also be a reflection of that higher EOR was more often possible for tumors in areas not located in proximity to these language and memory functions. A third explanation would be that a greater EOR reduced the necessity to proceed with adjunct oncological treatment and hence the potential negative cognitive effects of such treatments.

One major reason for the lack of significant associations between these potentially explanatory variables and cognitive dynamics could be that the overall treatment decisions are adapted to reduce the impact of, e.g., certain risk factors to influence the cognitive outcome variables. An eloquent tumor location may predispose for awake surgery leading to better surgical mapping and monitoring of these functions to define the functional surgical boundaries.

### Cognition and health-related quality of life—is there a connection?

Overall, our results suggest that both cognition and HRQoL deteriorated on a general level after glioma surgery at 3–5-month interval, but not concurrently for the same patients. There are several potential explanations. First, the cognitive tests were conducted by professionals, whereas the HRQoL-variables were patient-reported. It could be that lower cognitive skills reduced the patients’ capacity to more objectively assess their own situation, which might have attenuated any potential association. The results would perhaps be different if HRQoL had been assessed by a proxy or care personnel [16]. Second, the cognitive and HRQoL assessments were performed in temporal proximity, but not always on the very same day. The first months after surgery are intense and cognition and HRQoL could vary on a weekly basis, e.g., depending on temporal proximity to concurrent oncological treatments. Third, although cognitive dysfunction may well prevent patients to resume social and professional activities, the time frame around 3–5 months may be too early to differentiate patients in these HRQoL domains. This initial recovery period is often characterized by sick leave for most patients and particularly in case of adjunct oncological treatments. However, at 1 year postoperatively, approximately 70% had returned to work, which was fairly similar to previous reports (50–80%) [17, 20, 33, 37], although very high rates (near 100%) have been reported in some series [22]. Interestingly, a greater deterioration in attention and processing speed (coding) at 3 months was associated with a lower rate of return to work again within 1 year in our study. Such a deterioration could hence indicate a need for more rehabilitation and/or the necessity to consider an occupation change.

The only marginally significant finding detected between cognition and HRQoL was actually contrary to what was expected, i.e., that a larger deterioration in attention (digit span forward) and visual memory (RCFT copy) correlated with a lower rate of RP deterioration. We have no clear answer for this. It could be that a reduced attention span predisposes for a more optimistic appreciation of HRQoL or that this association was merely a statistical fluke. Interestingly, Nakajima and colleagues found that a deterioration in executive function was associated with lower GH, whereas lower postoperative verbal fluency correlated with lower RP and RE at 6 months postoperatively [20]. However, they used a different cognitive test battery, the follow-up was slightly later at 6 months postoperatively, and they evaluated HRQoL at 6 months rather than the dynamics between the pre- and postoperative state, which could explain the different results. However, similar to our study, Campanella and colleagues only found weak associations between cognitive variables and HRQoL [2], although they also used different test batteries and time intervals as compared to our study. In conclusion, larger prospective studies are needed to better understand these dynamics.

### Cognition and health-related quality of life—clinical implications and future directions

It is challenging to optimize the onco-functional balance in diffuse glioma patients. Any deterioration in sensorimotor, language, or cognitive function and HRQoL must be worth the gain in survival. When it comes to surgery, state of the art work-up currently includes radiological imaging with MRI, DTI, and possibly functional MRI to aid in preoperative surgical and awake surgery and neurophysiological monitoring may aid in defining the functional borders. However, whereas lower-order functions, e.g., sensorimotor, are relatively easy to monitor, the current challenge in this field is to better take into account higher-order cognitive functions [8]. This necessitates both a more in-depth neuroscientific understanding of the anatomo-functional circuits responsible for specific cognitive functions as well as development of techniques for awake monitoring of these complex, higher-order functions in the operating room [8, 12]. Furthermore, in addition to standard oncological follow-up of tumor recurrence, there is a need to follow-up on these patients regarding sensorimotor, language, and cognitive functions and HRQoL. Specifically, the role of active neurorehabilitation after glioma surgery has become an emerging area in recent years [44]. There are now several reports that cognitive rehabilitation both in the acute and outpatient setting, including retraining and functional compensation, promote functional reorganization and neuroplasticity that ultimately enhance cognitive recovery [44, 47]. In addition to traditional rehabilitation, there is an increased interest for adjunct therapies such as repetitive transcranial magnetic stimulation. This technique has initially been used to treat psychiatric conditions, but may also enhance neurological recovery after acute brain injury [4] as well as after brain tumor surgery [27]. Cognitive rehabilitation therapies are currently underutilized and deserve further interest in clinical care and research.

### Methodological considerations

The strength of this study is the combination of cognitive, language, and HRQoL data in a fairly sized study population, when compared to similar studies [2, 17, 20, 23, 31, 40, 45]. Since the available reports in the literature are still relatively limited, our results contribute with more data to deepen the understanding of the dynamics in cognition and HRQoL after diffuse glioma surgery.

There are also several limitations. First, similar to several previous reports [23, 40, 45], the main limitation was that the total patient cohort was still rather small and based on patients with heterogeneous tumors (primary/recurrence, type, and grade) and treatments (awake/asleep surgery and adjunct oncological treatment). The results should therefore be interpreted carefully. In future efforts, we aim to proceed with this data collection to provide more granular analyses of specific glioma patient sub-populations. Second, the time point of assessment is important. We chose around 3–5 months as a reasonable time to capture early recovery and before tumor recurrence typically takes place. However, many patients had received adjunct oncological treatment within this period, which obscured the results. Our findings illustrated that cognitive and HRQoL deteriorations are evident in the early course, but cognitive recovery may take at least 1 year [2, 36] and it would be interesting to further assess the longitudinal cognitive recovery at later time points as well. Third, we used a clinically feasible approach to study the association between cognitive dynamics after surgery in relation to relevant clinical variables. However, this approach may be considered not specific enough, taking into account the complexity of the glioma-brain interaction over time, neuroanatomy, and the spectrum of consequences due to surgical and oncological treatments [19]. Fourth, the raw scores of the cognitive tests were evaluated as *z*-scores. This enabled us to take into account age, sex, and the level of education of the individual patients. However, even when the best suited normative group is used, the validity of this may be questioned to some extent [32]. Particularly, the RCFT copy group exhibited a very narrow floor and roof for the *z*-score interval, which limited its usefulness. Lastly, practice effects are inevitable for the cognitive tests, as indicated by the RCFT copy and delayed recall tests. However, at least 3 months had passed between the two tests and we used randomization of words and figures in the tests as much as possible to reduce this effect.

## Conclusions

The major finding of this study is that whereas most patients recover from immediate postoperative neurological deficits, there is a concurrent decline in many cognitive and HRQoL domains for patients with diffuse gliomas 3 months following surgical treatment. The changes in cognitive test results were difficult to predict based on patient-, tumor-, and treatment-variables and were also only weakly correlated with HRQoL. This highlights the complexity in treating these patients and suggests that more extensive follow-ups and active rehabilitation may be warranted to improve recovery from this treatment intensive period for these patients.

## Supplementary Information

Below is the link to the electronic supplementary material.

Supplementary file1 (DOCX 12 KB)

Supplementary file2 (DOCX 13 KB)

Supplementary file3 (DOCX 148 KB)

Supplementary file4 (DOCX 30 KB)
