# Supplementary material for: Dynamics in cognition and health-related quality of life in grade 2 and 3 gliomas after surgery
Source: Acta Neurochir (Wien). 2022 Nov 4;164(12):3275–84. doi: 10.1007/s00701-022-05408-2 (PMC9705489; doi:10.1007/s00701-022-05408-2)
Supplement: Supplementary file 1 — Supplementary file1 (DOCX 12 KB) [file 701_2022_5408_MOESM1_ESM.docx]

**Supplementary Table 1. Battery of cognitive and language tests**

| **Tests** | **Cognitive and language functions** |
| --- | --- |
| TMT trial 2 | Visual attention and scanning |
| TMT trial 4 | Visual attention and scanning + mental flexibility and divided attention |
| Digit Span forward | Attention |
| Coding subtest from the Repeatable Battery for the Assessment of Neuropsychological Status | Attention and visuomotor processing speed |
| AVLT + delayed recall | Verbal learning + memory |
| RCFT copy + delayed recall | Visual construction, planning + visual memory |
| COWA | Verbal fluency |
| BNT | Confrontation naming, word finding |
