# Supplementary material for: Dynamics in cognition and health-related quality of life in grade 2 and 3 gliomas after surgery
Source: Acta Neurochir (Wien). 2022 Nov 4;164(12):3275–84. doi: 10.1007/s00701-022-05408-2 (PMC9705489; doi:10.1007/s00701-022-05408-2)
Supplement: Supplementary file 2 — Supplementary file2 (DOCX 13 KB) [file 701_2022_5408_MOESM2_ESM.docx]

**Supplementary Table 2. Health-related quality of life before and after glioma surgery**

| **RAND 36 variables** | **Preoperative** | **Postoperative** | **Paired difference ∆(postoperative-preoperative)** | **p-value** |
| --- | --- | --- | --- | --- |
| PF, median (IQR) | 95 (85-100) | 85 (71-95) | -5 (-19 – 4) | ***0.002*** |
| RP, median (IQR) | 100 (25-100) | 0 (0-75) | -25 (-100 – 0) | ***0.001*** |
| BP, median (IQR) | 90 (56-100) | 78 (45-100) | 0 (-23 – 10) | 0.30 |
| GH, median (IQR) | 65 (51-75) | 55 (45-70) | -10 (-20 – 5) | ***0.009*** |
| VT, median (IQR) | 58 (40-70) | 45 (35-65) | -5 (-20 – 5) | ***0.02*** |
| SF, median (IQR) | 75 (63-100) | 50 (38-88) | -25 (-25 – 0) | ***0.001*** |
| RE, median (IQR) | 67 (0-100) | 33 (0-100) | 0 (-33 – 33) | 0.66 |
| MH, median (IQR) | 72 (48-100) | 60 (52-84) | 0 (-12 – 8) | 0.42 |
