# Supplementary material for: Dynamics in cognition and health-related quality of life in grade 2 and 3 gliomas after surgery
Source: Acta Neurochir (Wien). 2022 Nov 4;164(12):3275–84. doi: 10.1007/s00701-022-05408-2 (PMC9705489; doi:10.1007/s00701-022-05408-2)
Supplement: Supplementary file 3 — Supplementary file3 (DOCX 148 KB) [file 701_2022_5408_MOESM3_ESM.docx]

**Supplementary figure 1. Flow chart for inclusion in the study.**


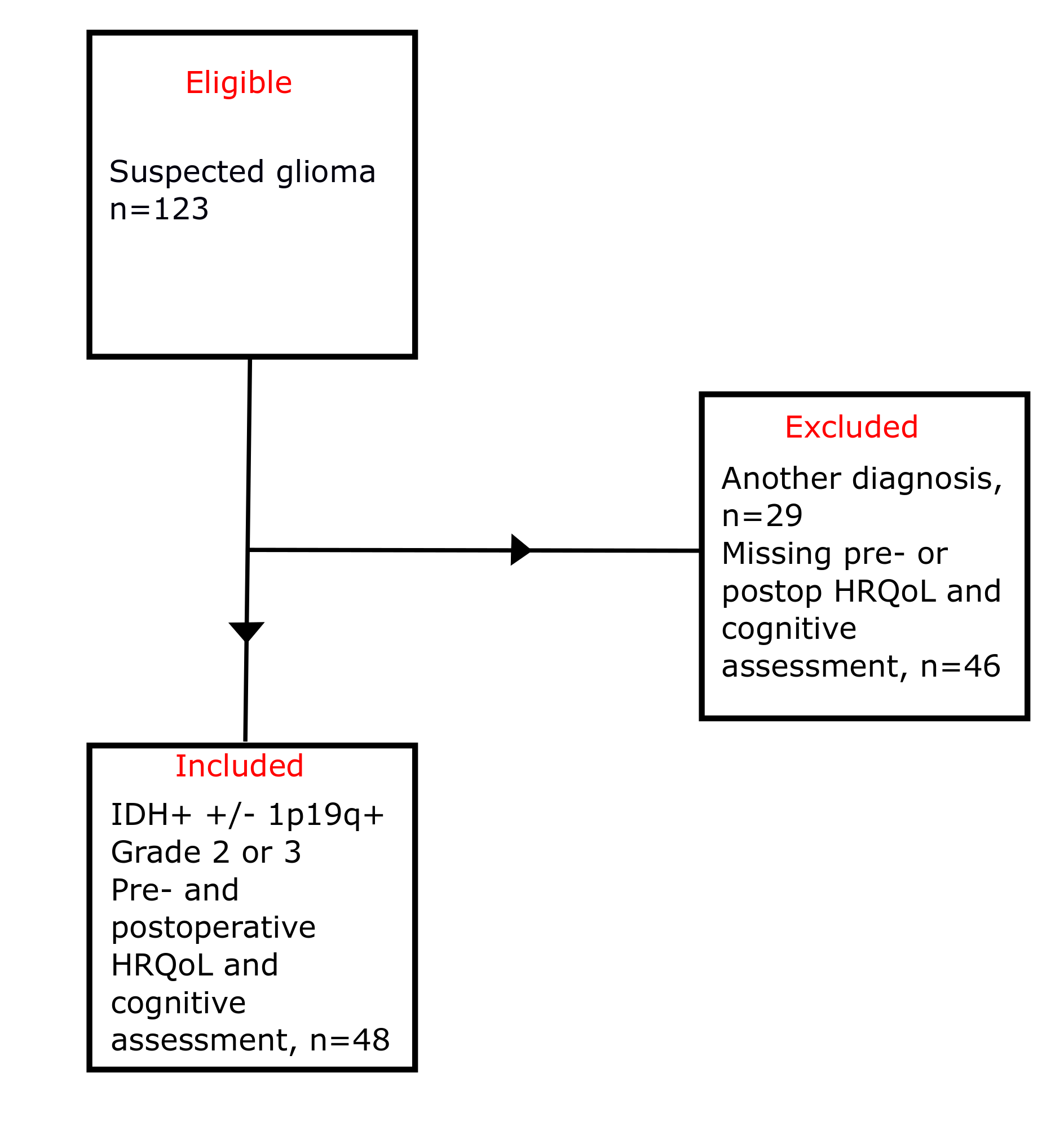


For the patients with “another diagnosis”, this included astrocytoma grade 4/glioblastoma (n=20), ganglioglioma (n=3), dysembryoplastic neuroepithelial tumor (n=1), neurocytoma (n=1), and unclear/other diagnosis than brain tumor (n=4)).
