# Supplementary material for: Dynamics in cognition and health-related quality of life in grade 2 and 3 gliomas after surgery
Source: Acta Neurochir (Wien). 2022 Nov 4;164(12):3275–84. doi: 10.1007/s00701-022-05408-2 (PMC9705489; doi:10.1007/s00701-022-05408-2)
Supplement: Supplementary file 4 — Supplementary file4 (DOCX 30 KB) [file 701_2022_5408_MOESM4_ESM.docx]

**Supplementary figure 2. A PCA loading plot of dynamic changes in cognition and HRQoL**


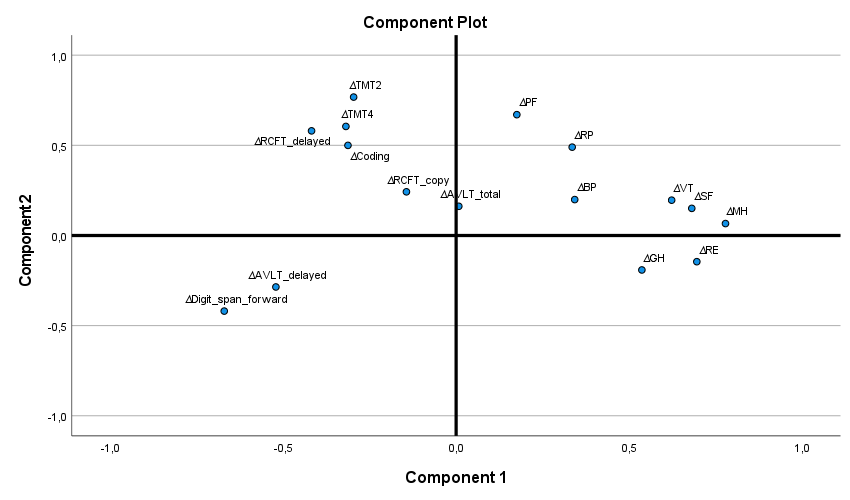


The PCA loading plot illustrates the association between the dynamics of cognitive test results and HRQoL before and after glioma surgery. It appeared that the dynamic changes in the various cognitive tests were associated with each other and that the dynamic changes in the various HRQoL domains were associated with each other, but that the cognitive and HRQoL-variables were more modestly associated with each other. The Kaiser-Meyer-Olkin test was only 0.26, indicating that the reliability of this analysis was low.
